# Antiretroviral stewardship in a tertiary academic hospital: The need for a clinical pharmacist

**DOI:** 10.4102/hsag.v28i0.2135

**Published:** 2023-08-30

**Authors:** Elmien Bronkhorst, Sonja Hattingh, Madan Poka

**Affiliations:** 1 School of Pharmacy, Sefako Makgatho Health Sciences University, Pretoria, South Africa

**Keywords:** HIV, antiretroviral stewardship, clinical pharmacist, antiretroviral therapy, Interventions, antiretroviral stewardship, clinical pharmacist, antiretroviral therapy

## Abstract

**Background:**

South Africa has the highest prevalence of people living with HIV globally. Although antiretroviral therapy provides solutions, evidence of antiretroviral resistance emerged, requiring the application of antiretroviral-stewardship programmes to curb medication-related problems.

**Aim:**

Identify and describe antiretroviral-stewardship pharmacist interventions in an active antiretroviral-stewardship programme.

**Setting:**

HIV-positive adults admitted to medical wards at a tertiary academic hospital in South Africa.

**Methods:**

A descriptive quantitative study was performed, utilising an antiretroviral-stewardship assessment tool to determine antiretroviral-related recommendations in the treatment of HIV-positive adults. The study employed purposive sampling. Treatment charts were evaluated to identify antiretroviral-stewardship recommendations. The number of recommendations highlighted the need for a clinical pharmacist in an active antiretroviral-stewardship programme. Descriptive data analysis with Pearson correlations was employed to display the data.

**Results:**

Medication-related problems were identified in 100% of study patients (*n* = 41), with an average of 2.46 interventions per patient. One-hundred-and-one medication-related problems were identified by using the antiretroviral-stewardship assessment tool. The identified problems included a lack of viral load testing (41, 100%), lack of CD4 count monitoring (15; 36.6%) and lack of prophylactic treatment against opportunistic infections (10; 24.4%). Medication-related problems included the presence of clinically significant drug–drug interactions and serious side effects, CD4 count decline despite being on antiretroviral therapy, unnecessary treatment interruptions including risk for IRIS, inappropriate antiretroviral therapy regimen, non-adherence and absence of treating tuberculosis as co-morbidity.

**Conclusion:**

Present study demonstrates the need of an active antiretroviral-stewardship programme’s benefits. The possible role of the clinical pharmacist as active participant and leader in this programme is highlighted.

**Contribution:**

Highlight the role of clinical pharmacists in antiretroviral stewardship.

## Introduction

Globally, around 37.9 million people are living with human immunodeficiency virus (HIV), of which 19.8% reside in South Africa (UNAIDS 2018a). South Africa homes around 8 million people living with HIV, of which 62% are treated with antiretroviral therapy (ART) (Stats SA 2018; UNAIDS 2018b). Antiretroviral therapy aims to suppress viral load, restore and conserve immunity, reduce HIV-related mortality, improve quality of life and prevent HIV transmission (Meintjes et al. 2017). South Africa has seen a decrease of 43% in acquired immunodeficiency syndrome (AIDS)-related deaths within the last decade as a result of the successful implementation of ART programmes (UNAIDS 2018a). This significant decline in mortality can be attributed to successful ART programmes, such as previously seen in countries like Brazil (Yager et al. 2017). Johnson et al. (2017) referenced several studies in South Africa to confirm the steady decline of mortality since the roll-out of a ART programme. Antiretroviral therapy’s success in the reduction of HIV-associated morbidity, mortality, transmission, stigma and improved quality of life obtained during the past two decades has been irrefutable. In spite of this significant reduction in morbidity and mortality, achieving the United Nations Programme on HIV/AIDS 95-95-95 targets will require vigilant viral load-informed care, especially to monitor resistance (Bain, Nkoke & Nouniap 2016). However, evidence of first-line treatment failure has emerged, calling for the utilisation of second-line therapy, which comes with greater cost implications and side effects (Schellack et al. 2017). In addition, the 90-90-90 goals introduced by the Joint United Nations Programme on HIV/AIDS (UNAIDS) and partners in 2014 (updated to 95-95-95 in 2020) have yet to be achieved. The target was to diagnose 95% of all HIV-positive individuals, to provide ART to 95% of those with a positive diagnosis and to obtain viral suppression in 95% of those treated with ART by 2030 (Bain et al. 2016). Unfortunately, this goal has not yet been reached. Antiretroviral therapy accounts for a major contribution to reach the 95-95-95 goals, and thus stewardship is of utmost importance (Bain et al. 2016).

The ARV treatment clinical guideline, which stipulates the latest treatment regimens, and when treatment in naïve patients should be deferred, was published by the National Department of Health in 2019. Treatment should be deferred in patients at risk for developing immune reconstitution inflammatory syndrome (IRIS), which is possible in treatment naïve patients, presenting with opportunistic infections and a low CD4 count (< 50 cells/µL) (NDoH 2019). This stipulates that all stable patients should be converted to a regimen containing dolutegravir and re-iterates that once treatment is started, it should not be interrupted (NDoH 2019).

Antiretroviral stewardship (ARV stewardship) should be included in existing antimicrobial stewardship programmes although challenges pertaining to antimicrobial stewardship still exist. Antiretroviral stewardship can, in addition to its place in antimicrobial stewardship, be defined as:

Co-ordinated interventions designed to improve continuity of care for patients receiving ARVs, through the utilisation of evidence-based ARV practices. This includes medication reconciliation, dosing, mitigation of drug interactions, and prevention of viral resistance. (Koren et al. 2020)

Antiretroviral-stewardship programmes are required to reduce the risk of medication-related errors and reduce treatment adverse effects in order to enhance patient safety (Koren et al. 2020; Meintjes et al. 2017). Furthermore, a focus on ARV stewardship can prevent virological resistance and optimise cost (Mehta et al. 2018). Numerous factors can be considered when it comes to ARV stewardship, such as adherence promotion, assessment of appropriateness of the current ART regimen and dosage, elimination of serious side effects and clinical significant drug–drug interactions (DDIs) while assessing cost effectiveness of treatment (Hsu et al. 2016). The most prevalent ARV-related errors encountered through several studies could be attributed to drug omission, incorrect dosing or dosing intervals and drug interactions. These ARV-related errors may cause the development of adverse effects, treatment failure or drug resistance, especially when they are not addressed as soon as possible (Koren et al. 2020). Implementation of a successful ARV-stewardship programme can assist South Africa in its battle against HIV (Yager et al. 2017); with the inclusion of a clinical pharmacist, a further reduction in post-prescription intervention was reported (Metha et al. 2018).

Morris et al. (2019) reported that interventions made by clinical pharmacists, as part of the interdisciplinary care team, contributed towards improved viral loads in paediatric patients. This statement can be justified by the significant increase of patients with an undetectable viral load based on interventions made by clinical pharmacists. These results can be concurred by another report, which found nearly 1500 clinical interventions made by pharmacists who cared for HIV-positive patients at a Veteran’s Affairs Medical Centre (VAMC) (Morris et al. 2019). Currently, there are no data available from South Africa that investigate the role of the clinical pharmacist in the utilisation of an ARV-stewardship programme.

In order to contribute to the awareness and implementation of ARV-stewardship programmes in South Africa, the present study was conducted among HIV-positive adults newly admitted to the internal medicine wards of a tertiary academic hospital in Gauteng. Even though hospitalisation rates among Persons Living With Human Immunodeficiency Virus (PLWH). have decreased because of effective ARV therapy, the potential for ARV-related errors persists during admission to and discharge from the hospital. Inpatients were exclusively included in this study, as the clinical pharmacists in the medical wards noticed that ART was not administered regularly, and adverse drug reactions and drug interactions were not monitored during admission. The objectives of the study were to identify ARV-stewardship interventions and describe the interventions performed by a pharmacist as part of an active ARV-stewardship programme. Through the interventions, the role of a clinical pharmacist in the utilisation of an ARV-stewardship programme will be described.

## Methods

### Study design and patient selection

A descriptive, quantitative study approach was employed. Participants who were considered to be providing the most relative information were selected purposively according to the inclusion criteria. Patient treatment records of HIV-positive adults over 18 years of age who were newly admitted to the internal medicine wards, regardless of the admission diagnosis, were included. The data collection period was 3 months (January to March 2020; period of assessment). Considering the prevalence of HIV-positive patients in South Africa is around 8% (Stats SA 2018; UNAIDS 2018b), this percentage was used to calculate the expected population size as 60. The calculated population size was based on the average admission rate in the medical ward, found to be 248 admissions per month. The sample size was calculated using Raosoft® with the 95% confidence interval and a margin of error of 3%. The expected sample size was calculated to be 60. During the study patient records that met the inclusion criteria were selected as part of the sampling criteria. The exclusion criteria pertained to treatment-naïve patients with opportunistic infections at risk for the development of IRIS, whose ARV treatment needs to be deferred. Records were evaluated to identify possible ARV-stewardship interventions using an ARV-stewardship assessment tool (ASAT) (Hsu et al. 2016).

A data collection instrument was designed as a tick-list, adapted from the validated instrument used in a study on ARV stewardship by Hsu et al. (2016). This ASAT form contained demographic data like the patient’s gender, age and co-morbidities. Antiretroviral-stewardship-related information included viral load, CD4 count, adherence, DDI’s, adverse effects, dosing, regime appropriateness and the need for prophylaxis. Drug–drug interactions were determined by using the HIV DDI calculator of Liverpool University available by accessing https://www.hiv-druginteractions.org/checker. The data collection instrument was piloted on five patients before the commencement of data collection. The data were not included in the final data set. The number of possible interventions identified was used to describe the need for a clinical pharmacist in ARV stewardship.

### Data collection and analysis

Data were collected by reviewing patient records, considering previous records pertaining to ART, retrospectively. Patient records were reviewed on a daily basis to identify current therapy problems. The researcher made recommendations to prescribers to resolve these identified problems; however, outcomes were not measured.

Data were analysed using Statistical Package for Social Science (SPSS) version 26. The frequency of data sets (demographic data, treatment regimens, DDIs and pharmacist recommendations) was analysed to obtain the number of patients and total percentage included for that specific data set. Cross-tabulation was done to obtain the number of patients and percentage of patients relevant to the specific data set. Pearson correlations with a *p* < 0.05 were performed.

### Ethical considerations

Ethical approval for this study was granted by the Sefako Makgatho University Research Ethics Committee (SMUREC) {SMUREC/P/235/2019:PG}, and permission to perform the study was obtained from the CEO of the tertiary academic hospital. All participant data was anonymised to ensure the confidentiality of patient information.

## Results

### Demographic data

The data of 41 patients were included in this study of which 61% (*n* = 25) were female. The mean age of the study population was 44.10 years, with the majority (26.8%) of the population in the age group 40–49 years (*n* = 11). Only six patients (14.6%) were in the age group of 18–29 years, and two (4.9%) were older than 65 years.

### Co-morbidities

A total of 19 patients (46.3%) presented with co-morbidities, with tuberculosis (TB) being the most prevalent condition (13; 31.7%). Other co-morbidities could be attributed to hypertension (5; 12.2%), diabetes mellitus (4; 9.8%), renal failure (3; 7.3%), chronic kidney disease (1; 2.4%), anaemia (1; 2.4%) and Herpes zoster (1; 2.4%). Out of the 13 patients diagnosed with TB, 11 (84.6%) received anti-tuberculosis treatment, either in the initial phase (9; 81.8%) or the continuation phase (2; 18.2%).

### Anti-retroviral stewardship assessment

**Viral load and CD4 count:** Viral load was recorded for only one patient (2.4%). A total of 29 patients’ (70.7%) CD4 counts were tested and recorded, of which 51.2% (21) were done upon admission. The cumulative percentage of those with a CD4 count below 200 cells/mm^3^ was 62.1% (18). Repeated CD4 counts identified six (14.6%) patients who experienced a decline in their immunological response, with three patients (7.3%) who experienced positive immunological responses. The main reason for the CD4 count decline was attributed to non-adherence reported by four patients who defaulted treatment.

**Antiretroviral therapy:** A total of 20 (48.8%) patients received first-line therapy, two (4.9%) received second-line therapy, four (9.8%) alternative regimens and 15 (36.6%) did not have their treatment regimen specified. Non-adherence was noted in five patients who reported defaulting therapy and required interventions on treatment adherence. Out of the total of 16 (39.0%) patients who experienced treatment interruptions, five patients defaulted treatment; three patients did not bring their medicine to the hospital; three patients posed at risk for IRIS; sickness complicated three patients’ treatment; while two patients had a regimen unknown to them and/or their prescriber. Seven of the treatment interruptions were documented to last less than 1 week, followed by four treatment interruptions continued for more than 3 weeks.

**Drug–drug interactions and side effects:** A total of seven patients (17.1%) were identified with different DDIs with clinical significance, in need of interventions. These interactions were identified as possible causative sub-therapeutic effects of certain drugs because of decreased concentrations or an increased risk for nephrotoxicity as presented in Table 1. A total of five (12.2%) patients experienced serious side effects of ART, which included kidney injury (4) and drug-induced liver injury (1). This could be attributed to an incorrect regimen, not in line with the National Treatment Guidelines (NDoH 2019), which requires interventions.

**TABLE 1:** Identified drug–drug interactions, which may influence the drug exposure.

Drug combination co-administered	Possible reason for concern	Proposed recommendations
**Interactions that increase drug exposure leading to increased risk of adverse effects**		
Vancomycin and tenofovir (TDF) (one patient)	Increased risk for nephrotoxicity. Both these agents are nephrotoxic and kidney function should be monitored.	Use an alternative option for vancomycin that is not nephrotoxic. If this is not possible, kidney function should be closely monitored.
Efavirenz (EFV) and morphine (one patient)	Increased morphine concentration with a higher risk for adverse effects related to morphine.	Tramadol may be a safer alternative to morphine as its potential risk for opioid toxicity is weaker.
**Interactions that decrease drug exposure lead to decreased drug efficacy and/or possible ARV resistance**		
Rifampicin and dolutegravir (DTG) (one patient)	Dolutegravir exposure is decreased and thus increased risk for ARV resistance and decrease ARV efficacy	Increase dolutegravir to 50 mg twice daily in adult patients.
Efavirenz (EFV) and carbamazepine (one patient)	Decreased carbamazepine concentration	Alternative anticonvulsant therapy should be considered. Safe options include valproate, gabapentin, pregabalin, topiramate and vigabatrin.
Lopinavir (LPV) and clopidogrel† (one patient)	Decreased clopidogrel concentration	This combination should be avoided. Safe alternatives include aspirin, enoxaparin, fondaparinux, heparin and prasugrel.
Rifampicin and lopinavir/rotinavir (LPV/r)† (one patient)	Decreased lopinavir concentration and thus decreased ARV exposure	Adequate exposure to LPV/r may be obtained through the administration of a higher dose LPV/r (400/400 mg twice a day). This dose is associated with an increased risk for liver and gastrointestinal toxicity, justifying the fact that co-administration should be avoided.
Rifampicin and nevirapine† (one patient)	Decreased nevirapine concentration and thus decreased ARV exposure	Rifabutin can be used as an alternative to rifampicin. Alternatively, nevirapine’s dose can be increased by 50% to 300 mg twice a day, but no safety data is available and careful monitoring will be required.

† , contraindicated combination.

**Prophylaxis against opportunistic infections:** Out of the 18 patients with a CD4 count of less than 200 cells/mm^3^, only eight (44.4%) received co-trimoxazole as either prophylaxis (4) or treatment (4).

**FIGURE 1:**
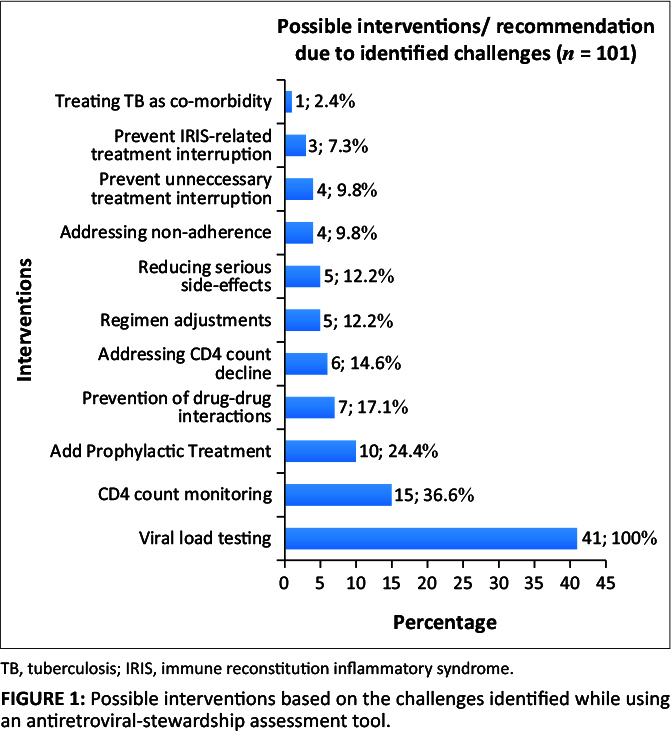
Possible interventions based on the challenges identified while using an antiretroviral-stewardship assessment tool.

**Identified problems requiring interventions/recommendation:** The total study population of 41 patients (100%) required interventions. A total of 101 opportunities for interventions/recommendations could be identified using the ASAT. An average of 2.46 (SD = ±15.24) medication-related problems per patient requiring interventions was identified. Three patients (7.3%) required a maximum of five interventions, and seven (17.1%) patients required only one intervention. Although no statistically significant correlations were found, the majority of interventions required could be attributed to lack of viral load testing (41; 100%), lack of CD4 count monitoring (15; 36.6%) and omitted prophylactic treatment against opportunistic infections (10; 24.4%) (see Figure 1).

**FIGURE 2:**
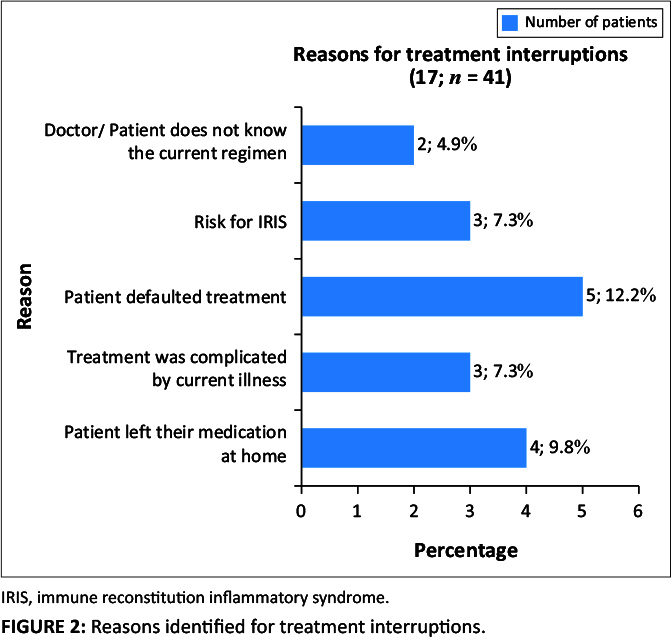
Reasons identified for treatment interruptions.

Treatment interruptions were identified as a challenge concerning ARV stewardship, encountered by 17 patients (41.5%). These interruptions were based on different reasons as presented in Figure 2, with the leading cause being patients who defaulted treatment.

## Discussion

Globally, out of the 38 million people living with HIV, approximately 50.5% are female. In South Africa, women predominate these statistics even further with a HIV-positive prevalence of 62.67% (UNAIDS 2018a, 2018b). Similarly, this study determined that HIV-positive females are more predominant in numbers, contributing to 61% of the population. Female health-seeking behaviour may contribute to this phenomenon (UNAIDS 2018b).

This study showed that viral load testing and monitoring do not form part of the routine follow-up and monitoring of HIV-positive patients on ART. According to the latest National Treatment Guidelines from the National Department of Health (NDoH) in South Africa, viral load testing should be done prior to ART initiation, then 6 months after ART initiation and then every 6 months thereafter (Meintjes et al. 2017; NDoH 2019). This is necessary to prompt the determination of virological response to ART in order to detect adherence problems and treatment failure timeously. No patient had a viral load test within 6 months and thus 100% of the study population required this intervention. This finding suggests that the National Treatment Guidelines (NDoH 2019) are not adhered to, and that there is a definite need for intervention. A similar finding was observed in a study done in Indonesia, where they found the lack of viral load testing the major reason for underdiagnoses of treatment failure, thus vitalising viral load testing as part of optimal ART (Limmade et al. 2019). Viral load testing is of utmost importance in determining the progress of treatment, identifying therapy failure and evaluating the risk for transmission. Meka et al. (2020) found the barriers to viral load testing to be both at health care system and patient level. Ignorance, stock shortages of tests and staff shortages among others were identified as barriers to viral load testing (Meka et al. 2020). Furthermore, the NDoH guidelines only describe the use of CD4 count monitoring to determine susceptibility to opportunistic infections and the need for possible prophylactic treatment (NDoH 2019). Thus, CD4 count monitoring by no means carries any significance in determining ART treatment success. Another study stated that viral load testing should be classified as highly important so that sub-optimal adherence can be identified as quickly as possible to address possible drug resistance and viral transmission at an early stage and to improve virological outcomes (Limmade et al. 2019).

Meintjes et al. (2017) stated that ART failure can be declared once the viral load rises above 1000 copies/mL on two consecutive measurements taken 2–3 months apart. Once the latter has been confirmed as an ART failure, it is recommended to switch the patient to a second-line regimen. This study found 14.6% of patients on either second-line therapy or another regimen (9.8%), without having viral load tested first. The most common reason given for ART failure was non-adherence (Meintjes et al. 2017). Gupta and Das (2019), identified the ever-growing population as a major hindrance to treatment adherence because of limited accessibility to health care services. Furthermore, the stigma associated with a positive diagnosis of HIV leads to additional hindering factors such as a lack of support from family and friends (Gupta & Das 2019). The fact that currently preferred ART regimens are less complex, better tolerated and less toxic is expected to eliminate the primary adherence barriers identified as complicated regimens and unpleasant feeling, by Sauceda et al. (2017).

Two studies found no mortality because of IRIS, after early initiation of ART, including in ARV-naïve patients (Blanc et al. 2011; Demitto et al. 2019). The present study reported treatment interruptions because of the possible risk for IRIS (3, 7.3%), which can be regarded as unnecessary treatment interruptions based on the above-mentioned finding. Furthermore, the National Treatment Guidelines explicitly state that treatment, once started, should not be interrupted (NDoH 2019). This notion can further be supported by evidence from the same studies that used early survival analysis and found no risk for early mortality in ARV-experienced patients who have IRIS, alongside a high baseline HIV viral load (Demitto et al. 2019). The need for viral load testing is further stressed and the assumption for the risk of IRIS is further queried.

Untreated HIV is associated with decreased CD4 counts and increased viral loads (Kufa et al. 2019). However, the present study found a CD4 count decline in six patients while being treated with ART. This may indicate poor adherence, inappropriate regimen (because of incorrect drugs or incorrect dosing), DDIs that decrease ARV exposure or ARV resistance. Kufa et al. (2019) suggest that CD4 count should be measured 12 months after ART initiation in patients who had a baseline CD4 count of less than 200 cells/mm^3^. Continued measuring is then recommended at 12 monthly intervals until a CD4 count above 200 cells/mm^3^ is maintained (Kufa et al. 2019).

CD4 count monitoring carries weight in the decision-making process of initiating prophylaxis against opportunistic infections. Secondary prophylaxes with antimicrobial agents are indicated in patients with a CD4 count below 200 cells/mm^3^ (Maartens 2018). Although most of the patients had an available CD4 count tested within a 6-month period (26; 63.4%), many of them did not receive co-trimoxazole prophylactic treatment (10) against opportunistic infections, such as *Pneumocystis jirovecii* pneumonia.

Billedo, Berkowitz and Cha (2015) found that two out of every five patients admitted to the hospital require interventions pertaining to incorrect dosing and clinically significant DDIs. This was concurred by a study with similar results demonstrating a 33% incidence of incorrect dosing alongside DDIs with clinical significance (Bias et al. 2014). This study found similar results with 29.3% of patients who received an inappropriate ART regimen, with or without the presence of DDIs carrying clinical significance. Drug–drug interactions may give rise to increased drug exposure and lead to the frequent emergence of adverse effects. Alternatively, these DDIs may reduce ARV exposure leading to potential ARV resistance (Patel et al. 2018).

Billedo et al. (2015) could intervene on 98% of medication-related errors identified by the ARV-stewardship programme. They concluded on the importance of an active ARV-stewardship programme that includes ARV restriction and daily monitoring by a clinical pharmacist, in order to prevent medication errors and improve safety in HIV-positive patients (Bias et al. 2014; Billedo et al. 2015). The ASAT used in this study allowed the identification of a number of challenges that may require interventions in order to steward ART. These results suggest the need of an active ARV-stewardship programme, which may contribute to findings similar to those obtained in the mentioned study. Good adherence to ART enhances the overall health status, survival and quality of life of patients living with HIV/AIDS (Limmade et al. 2019). As many of the identified challenges may hinder adherence, it can be concluded that the implementation of these interventions identified through an active ARV-stewardship programme may promote adherence and prevent treatment interruptions, which will ultimately improve patient health. Taking all the factors of an ARV-stewardship programme into consideration, the authors recommendation for an active ARV-stewardship programme is strengthened.

## Limitations

The findings of this study relied on purposive sampling, including only medical wards of one hospital, and thus, the results cannot be generalised. Furthermore, because of the COVID-19 pandemic and limited patient admissions, the sample size is smaller than the intended sample size. This study is only intended to identify interventions in order to evaluate the role of an active ARV-stewardship programme and not monitoring the outcomes of interventions. This study did not examine possible reasons for non-adherence and whether previous interventions to boost adherence were implemented. As the outcomes of interventions were not monitored, their cost implications could not be monitored either.

## Contribution

The authors recommend the development and implementation of an active ARV-stewardship programme in all hospitals. The addition of an active ARV-stewardship programme will help to identify medication-related problems that require intervention and allow optimal ART to patients. The addition of the clinical pharmacist in ARV-stewardship programmes may carry a significant impact in the quantity and most importantly the quality of interventions. However, the capacity and knowledge of the clinical pharmacist regarding ARV-stewardship programmes may be researched in the future. This statement can be further supported by future research on the impact of implemented interventions from a clinical pharmacist on ARV stewardship.

## Conclusion

This study supports the need for the implementation of an active ARV-stewardship programme. The results found in this study clearly highlight the immense role that an active ARV-stewardship programme will have pertaining to viral load testing and monitoring, and CD4 count monitoring to evaluate the need for initiation of anti-microbial prophylaxis. Patient regimen monitoring relating to drug selection and dose prescribed, identifying and addressing non-adherence, preventing clinically significant DDIs and thereby reducing the risk for adverse effects or ARV resistance and prevention of unnecessary treatment interruptions can be beneficial. Although these parameters are not exclusive, they were mainly identified as reasons for the inclusion of possible interventions.
